# (3*R*,5*S*)-5(3)-Carb­oxy-3,4,5,6-tetra­hydro-2*H*-1,4-thia­zin-4-ium-3(5)-carboxyl­ate

**DOI:** 10.1107/S1600536808005151

**Published:** 2008-02-27

**Authors:** Gustavo Portalone, Alberto Cassetta, Marcello Colapietro, Susanne Heidi Plattner

**Affiliations:** a Chemistry Department, University of Rome I "La Sapienza", P.le A. Moro 5, I-00185 Rome, Italy; b Institute of Crystallography, CNR, Trieste Outstation, Area Science Park-Basovizza, S.S.14 Km 163.5, I-34012 Trieste, Italy

## Abstract

The molecule of the zwitterionic title compound, C_6_H_9_NO_4_S, which lies on a mirror plane, shows a puckered chair conformation of the six-membered ring with the S and N atoms out of the mean plane of the other four C atoms by 0.929 (2) and 0.647 (2) Å, respectively. The ionized carboxyl group is equatorially oriented. The hydrogen-bonding network includes very short O—H⋯O [2.470 (2) Å] and N—H⋯S [3.471 (2) and 3.416 (2) Å] inter­molecular contacts.

## Related literature

For the detection of 1,4-thio­morpholine-3,5-dicarboxylic acid (THT) as a normal component in bovine brains and human urine, see: Cavallini, Pecci *et al.* (1985 ▶); Cavallini, Matarese *et al.* (1985 ▶); Matarese *et al.* (1987 ▶); Cavallini *et al.* (1991 ▶). For the previous structure determination of the (3*R*,5*R*) epimer of THT, see: Portalone *et al.* (1993 ▶). For related literature, see: Allen *et al.* (1997 ▶); Paglialunga Paradisi *et al.* (1990 ▶).
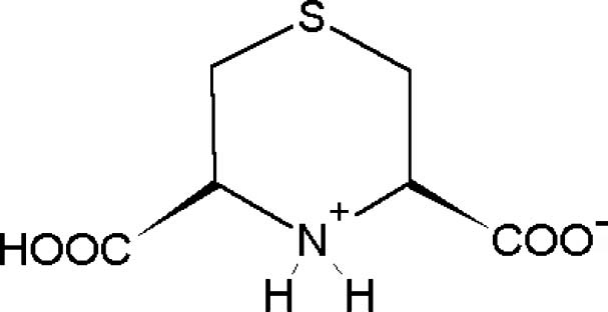


## Experimental

### 

#### Crystal data

C_6_H_9_NO_4_S
*M* *_r_* = 191.21
Orthorhombic, 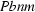

*a* = 6.1641 (8) Å
*b* = 9.323 (1) Å
*c* = 12.760 (1) Å
*V* = 733.29 (14) Å^3^
*Z* = 4
Mo *K*α radiation
μ = 0.41 mm^−1^
*T* = 298 (2) K
0.20 × 0.15 × 0.10 mm

#### Data collection

Huber CS four-circle diffractometer
Absorption correction: ψ scan (North *et al.*, 1968 ▶) *T*_min_ = 0.916, *T*_max_ = 0.958
1840 measured reflections
1060 independent reflections
998 reflections with *I* > 2σ(*I*)
*R*_int_ = 0.02
3 standard reflections every 97 reflections intensity decay: 2%

#### Refinement

*R*[*F* ^2^ > 2σ(*F* ^2^)] = 0.032
*wR*(*F* ^2^) = 0.094
*S* = 1.07
1060 reflections
76 parameters
H atoms treated by a mixture of independent and constrained refinement
Δρ_max_ = 0.25 e Å^−3^
Δρ_min_ = −0.28 e Å^−3^

### 

Data collection: *XCS* (Colapietro *et al.*, 1992 ▶); cell refinement: *XCS*; data reduction: *XCS*; program(s) used to solve structure: *SIR97* (Altomare *et al.*, 1999 ▶); program(s) used to refine structure: *SHELXL97* (Sheldrick, 2008 ▶); molecular graphics: *WinGX* (Farrugia, 1999 ▶); software used to prepare material for publication: *WinGX*.

## Supplementary Material

Crystal structure: contains datablocks global, I. DOI: 10.1107/S1600536808005151/rz2198sup1.cif

Structure factors: contains datablocks I. DOI: 10.1107/S1600536808005151/rz2198Isup2.hkl

Additional supplementary materials:  crystallographic information; 3D view; checkCIF report

## Figures and Tables

**Table 1:** Hydrogen-bond geometry (Å, °)

*D*—H⋯*A*	*D*—H	H⋯*A*	*D*⋯*A*	*D*—H⋯*A*
O1—H1⋯O1^i^	1.24	1.24	2.4704 (19)	180
N4—H41⋯S1^ii^	0.87 (3)	2.60 (3)	3.4713 (15)	179 (3)
N4—H42⋯S1^iii^	0.80 (3)	2.72 (3)	3.4155 (16)	147 (3)

Symmetry codes: (i) 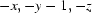; (ii) 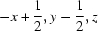; (iii) 
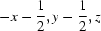
.

**Fig. 1.**
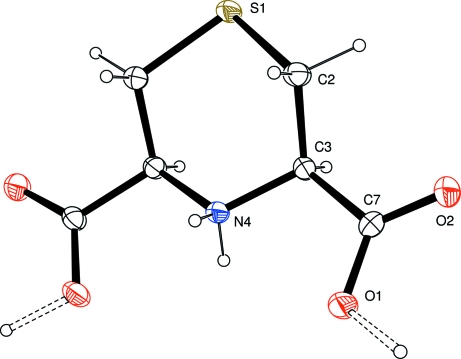
The molecular component in the title compound showing the zwitterion lying on a crystallographic mirror plane and the atom-labelling scheme. Displacement ellipsoids are drawn at the 50% probability level and H atoms are shown as small spheres of arbitrary radii.

**Fig. 2.**
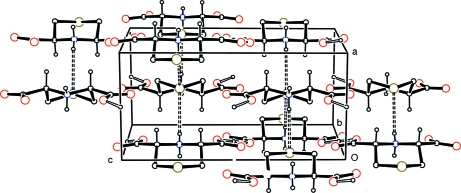
Packing diagram of the title compound viewed approximately down the a axis. H atoms are shown as small spheres of arbitrary radii. For the sake of clarity, H21, H22, H41 and H42 are omitted. H bonding is indicated by dashed lines.

## supplementary crystallographic information

### Comment

The detection of 1,4-thiomorpholine-3,5-dicarboxylic acid (THT) as normal component in bovin brain (Cavallini, Pecci *et al.*, 1985) and human urine (Matarese *et al.*, 1987) has stimulated the investigation of the biological role played by this unusual cyclic, sulfur containing imino acid (Cavallini *et al.*, 1991). Here we report the *x*-ray structure determination of the (3*R*,5S) epymer (THTC). The asymmetric unit of the title compound comprises a half-zwitterion disposed about a mirror plane along the line joining atoms S1 and N4 and perpendicular to the plane formed by C2, C3, C2^ì^ and C3^ì^ [symmetry code: (i) *x*, *y*, -*z* + 1/2]. From Fig. 1 it appears that the six-membered ring adopts a puckered chair conformation with the carboxyl group in equatorial position. The hydrogen-bonding network (Fig. 2) includes very short O—-H···O and N—-H···S (Allen *et al.*, 1997) intermolecular contacts (Table 1).

### Experimental

(3*R*,5S)-tetrahydro-2*H*-1,4-thiazine-3,5-dicarboxylic acid was obtained as described previously (Paglialunga Paradisi *et al.*, 1990). Crystals were grown from a water solution (0.1 mmol in *ca* 6 ml) by slow evaporation of the solvent.

### Refinement

All H atoms were found in a difference Fourier map. Positional and thermal parameters of all H atoms but H1, which lies in special position and for which U_iso_ value was set equal to 2.0 U_eq_(O1), were refined isotropically.

### Crystal data

**Table:**

C_6_H_9_NO_4_S	*F*_000_ = 400
*M**_r_* = 191.21	*D*_x_ = 1.732 Mg m^−^^3^
Orthorhombic, *P**b**n**m*	Mo *K*α radiation λ = 0.71069 Å
Hall symbol: -P 2c 2ab	Cell parameters from 87 reflections
*a* = 6.1641 (8) Å	θ = 20–25º
*b* = 9.323 (1) Å	µ = 0.41 mm^−^^1^
*c* = 12.760 (1) Å	*T* = 298 (2) K
*V* = 733.29 (14) Å^3^	Block, colourless
*Z* = 4	0.20 × 0.15 × 0.10 mm

### Data collection

**Table:**

Huber CS four-circle diffractometer	*R*_int_ = 0.02
Radiation source: fine-focus sealed tube	θ_max_ = 30.0º
Monochromator: graphite	θ_min_ = 3.2º
*T* = 298(2) K	*h* = 0→8
ω scans	*k* = 0→13
Absorption correction: ψ scan(North *et al.*, 1968)	*l* = 0→17
*T*_min_ = 0.916, *T*_max_ = 0.958	3 standard reflections
1840 measured reflections	every 97 reflections
1060 independent reflections	intensity decay: 2%
998 reflections with *I* > 2σ(*I*)	

### Refinement

**Table:**

Refinement on *F*^2^	Secondary atom site location: difference Fourier map
Least-squares matrix: full	Hydrogen site location: inferred from neighbouring sites
*R*[*F*^2^ > 2σ(*F*^2^)] = 0.032	H atoms treated by a mixture of independent and constrained refinement
*wR*(*F*^2^) = 0.094	*w* = 1/[σ^2^(*F*_o_^2^) + (0.064*P*)^2^ + 0.2054*P*] where *P* = (*F*_o_^2^ + 2*F*_c_^2^)/3
*S* = 1.07	(Δ/σ)_max_ < 0.001
1060 reflections	Δρ_max_ = 0.25 e Å^−^^3^
76 parameters	Δρ_min_ = −0.28 e Å^−^^3^
Primary atom site location: structure-invariant direct methods	Extinction correction: none

### Special details

**Table:**

Geometry. All e.s.d.'s (except the e.s.d. in the dihedral angle between two l.s. planes) are estimated using the full covariance matrix. The cell e.s.d.'s are taken into account individually in the estimation of e.s.d.'s in distances, angles and torsion angles; correlations between e.s.d.'s in cell parameters are only used when they are defined by crystal symmetry. An approximate (isotropic) treatment of cell e.s.d.'s is used for estimating e.s.d.'s involving l.s. planes.
Refinement. Refinement of *F*^2^ against ALL reflections. The weighted *R*-factor *wR* and goodness of fit *S* are based on *F*^2^, conventional *R*-factors *R* are based on *F*, with *F* set to zero for negative *F*^2^. The threshold expression of *F*^2^ > σ(*F*^2^) is used only for calculating *R*-factors(gt) *etc*. and is not relevant to the choice of reflections for refinement. *R*-factors based on *F*^2^ are statistically about twice as large as those based on *F*, and *R*- factors based on ALL data will be even larger.

### Fractional atomic coordinates and isotropic or equivalent isotropic displacement parameters (Å^2^)

**Table:**

	*x*	*y*	*z*	*U*_iso_*/*U*_eq_	
S1	0.04543 (7)	0.05914 (4)	0.2500	0.01738 (15)	
O1	−0.04198 (17)	−0.42620 (10)	0.07779 (8)	0.0235 (2)	
H1	0.0000	−0.5000	0.0000	0.049*	
O2	−0.04168 (16)	−0.23791 (11)	−0.03208 (7)	0.0233 (2)	
N4	−0.0505 (2)	−0.27615 (15)	0.2500	0.0152 (3)	
H41	0.077 (5)	−0.316 (4)	0.2500	0.046 (9)*	
H42	−0.129 (5)	−0.344 (3)	0.2500	0.035 (7)*	
C2	0.0797 (2)	−0.06777 (12)	0.14454 (10)	0.0191 (3)	
H21	0.232 (3)	−0.105 (2)	0.1441 (12)	0.031 (4)*	
H22	0.054 (3)	−0.012 (2)	0.0759 (16)	0.029 (5)*	
C3	−0.08019 (18)	−0.19183 (12)	0.15032 (9)	0.0154 (2)	
H3	−0.232 (3)	−0.1555 (18)	0.1489 (11)	0.019 (4)*	
C7	−0.05172 (18)	−0.29148 (12)	0.05503 (10)	0.0167 (2)	

### Atomic displacement parameters (Å^2^)

**Table:**

	*U*^11^	*U*^22^	*U*^33^	*U*^12^	*U*^13^	*U*^23^
S1	0.0253 (2)	0.0108 (2)	0.0160 (2)	−0.00097 (13)	0.000	0.000
O1	0.0403 (6)	0.0134 (4)	0.0170 (4)	0.0028 (3)	0.0010 (4)	−0.0023 (3)
O2	0.0352 (5)	0.0204 (5)	0.0142 (4)	−0.0015 (4)	−0.0003 (3)	0.0006 (3)
N4	0.0220 (7)	0.0109 (6)	0.0127 (6)	−0.0010 (5)	0.000	0.000
C2	0.0275 (6)	0.0142 (5)	0.0156 (5)	−0.0037 (4)	0.0027 (4)	−0.0009 (4)
C3	0.0211 (5)	0.0126 (4)	0.0124 (5)	0.0003 (4)	−0.0012 (4)	0.0006 (4)
C7	0.0198 (5)	0.0156 (5)	0.0146 (5)	−0.0008 (4)	−0.0011 (4)	−0.0026 (4)

### Geometric parameters (Å, °)

**Table:**

S1—C2	1.8043 (12)	N4—H41	0.87 (3)
S1—C2^i^	1.8043 (12)	N4—H42	0.80 (3)
O1—C7	1.2905 (14)	C2—C3	1.5211 (16)
O1—H1	1.2352	C2—H21	1.00 (2)
O2—C7	1.2201 (16)	C2—H22	1.03 (2)
N4—C3^i^	1.5064 (13)	C3—C7	1.5403 (16)
N4—C3	1.5064 (13)	C3—H3	0.997 (17)
			
C2—S1—C2^i^	96.46 (8)	S1—C2—H22	106.5 (11)
C7—O1—H1	111.77	H21—C2—H22	108.4 (13)
C3^i^—N4—C3	115.20 (12)	N4—C3—C2	111.04 (10)
C3^i^—N4—H41	109.5 (10)	N4—C3—C7	109.75 (9)
C3—N4—H41	109.5 (10)	C2—C3—C7	110.28 (9)
C3^i^—N4—H42	109.9 (9)	N4—C3—H3	107.9 (8)
C3—N4—H42	109.9 (9)	C2—C3—H3	110.5 (10)
H41—N4—H42	102 (3)	C7—C3—H3	107.3 (9)
C3—C2—S1	112.75 (8)	O2—C7—O1	126.95 (11)
C3—C2—H21	110.2 (12)	O2—C7—C3	118.55 (11)
S1—C2—H21	109.9 (10)	O1—C7—C3	114.50 (10)
C3—C2—H22	108.9 (10)		
			
C2^i^—S1—C2—C3	56.74 (12)	N4—C3—C7—O2	−170.39 (11)
C3^i^—N4—C3—C2	59.40 (16)	C2—C3—C7—O2	−47.76 (14)
C3^i^—N4—C3—C7	−178.42 (8)	N4—C3—C7—O1	9.90 (14)
S1—C2—C3—N4	−61.40 (12)	C2—C3—C7—O1	132.53 (11)
S1—C2—C3—C7	176.73 (8)		

Symmetry codes: (i) *x*, *y*, −*z*+1/2.

### Hydrogen-bond geometry (Å, °)

**Table:**

*D*—H···*A*	*D*—H	H···*A*	*D*···*A*	*D*—H···*A*
O1—H1···O1^ii^	1.24	1.24	2.4704 (19)	180.00 (9)
N4—H41···S1^iii^	0.87 (3)	2.60 (3)	3.4713 (15)	179 (3)
N4—H42···S1^iv^	0.80 (3)	2.72 (3)	3.4155 (16)	147 (3)

Symmetry codes: (ii) −*x*, −*y*−1, −*z*; (iii) −*x*+1/2, *y*−1/2, *z*; (iv) −*x*−1/2, *y*−1/2, *z*.
